# Review of the mite genus *Krantzolaspina* Datta & Bhattacharjee (Mesostigmata, Parholaspididae) with re-description of *K. angustatus* comb. nov. (Ishikawa) from Indonesia

**DOI:** 10.3897/zookeys.997.54262

**Published:** 2020-11-25

**Authors:** Edwin Javier Quintero-Gutiérrez, Dorothee Sandmann, Orlando Cómbita-Heredia, Bernhard Klarner, Rahayu Widyastuti, Stefan Scheu

**Affiliations:** 1 Instituto Colombiano Agropecuario (ICA), Subgerencia de Análisis y Diagnóstico, Grupo Red de Análisis y Diagnóstico Fitosanitario, Laboratorio de Análisis y Diagnóstico Fitosanitario de Manizales, Caldas, Carrera 30 # 65-15, Manizales, Colombia; 2 University of Göttingen, J.F. Blumenbach Institute of Zoology and Anthropology, Untere Karspüle 2, 37073 Göttingen, Germany; 3 Acarology Laboratory, Ohio State University, 1315 Kinnear Rd., Columbus, OH 43212, USA; 4 Centro de Inversitgación en Acarología CIACARI Bogota, Colombia; 5 Institut Pertanian Bogor, Department of Soil Science and Land Resources, Damarga Campus, Bogor 16680, Indonesia; 6 University of Göttingen, Centre of Biodiversity and Sustainable Land Use, Büsgenweg 1, 37077 Göttingen, Germany

**Keywords:** Gamasina, monotype, morphology, oriental region, rainforest, tropical lowland

## Abstract

Herein, we update the diagnosis and description of the genus *Krantzolaspina* Datta & Bhattacharjee and provide a list of the three valid species including new combinations and synonyms, as follows: 1) *Krantzolaspina angustatus* (Ishikawa, 1987) **comb. nov.** (= *Indutolaelaps jiroftensis*Hajizadeh et al., 2017**syn. nov**.), 2) *K. rebatii* Datta & Bhattacharjee, 1989 and 3) *K. solimani* (Metwali, 1983) **comb. nov.** Finally, we re-describe *K. angustatus* (Ishikawa, 1987) **comb. nov.** based on the holotype from Japan, voucher specimens from Iran and additional females that we found in soil samples from oil palm plantations in Sumatra, Indonesia.

## Introduction

The mesostigmatic mite family Parholaspididae has a total of 14 known genera distributed worldwide (Marchenko 2016). Species of Parholaspididae are found in a variety of habitats, such as soil-litter of forests and disturbed habitats (Jung et al. 2010), dead wood, moss, tree holes and some have been found associated with mammal nests, such as Cricetidae and Muridae (Yin et al. 1964, Petrova 1967a, 1967b, Gu 1984, Chen et al. 1994, Gu and Guo 1996). Other species have been reported from caves (Willmann 1940, Ishikawa 1995a, 2002) and also as early colonisers after habitat disturbance.

Indonesia is a biodiversity hotspot (Rintelen et al. 2017). However, knowledge on most groups of mites is still very limited. For instance, the family Parholaspididae is represented by only two described species (*Holaspulus viduus* Berlese, 1905 and *Parholaspis desertus* Berlese, 1918; both from Java). This is comparatively low with some countries in the Oriental region that have many more species records of Parholaspididae, such as China (56 species), Japan (28), Philippines (9) and Malaysia (7) (Berlese 1905, 1918, Vitzthum 1926, Evans 1956, Krantz 1960, Yin et al. 1964, 1999, Ishikawa 1966, 1969, 1976, 1979, 1980a, 1980b, 1987a, 1987b, 1993, 1994, 1995a, 1995b, 2002, Petrova and Tascaeva 1968, Bhattacharyya 1977, Gu 1984, Datta and Bhattacharjee 1989, Liang and Hu 1993, Tseng 1993, Yin and Bei 1993, Chen et al. 1994, Lee and Cho 1995, Gu and Guo 1996, Lee and Lee 1996a, 1996b, 2000, Ishikawa and Saichuae 1997, Ma 1998, 2004, 2010, 2012, Ma and Yin 1999, Ma and Yan 2001, Bei et al. 2004, 2009, Ma and Lin 2006, 2011, 2013, 2014a, 2014b, 2015, Bai and Ma 2014, Bai et al. 2014, Lee 2014, Kontschaán et al. 2015, Bhattacharyya and Kheto 2016). Other countries in the region have few species recorded but that may merely indicate a lack of knowledge: India (3 species), the Korean peninsula (1) and Thailand (1).

Despite the remarkable faunal diversity of the oriental region and numerous reported species of parholaspidid mites, there are shortcomings in the old species descriptions (lack of leg chaetotaxy, information on external poroidotaxy and adenotaxy of the idiosoma). For the previous, we decided to add information and organised the monotypic genus *Krantzolaspina*, based on available specimens, photographs from paratypes, original description and illustrations. For this reason, in the present work, a re-description of *Krantzolaspina angustatus* comb. nov. based on holotype, review of the generic concept based on type material and literature, a new synonymy and a list of valid species with some comments is presented.

### Material and methods

This study forms part of an investigation on soil and canopy arthropods of rainforests and agricultural systems in Jambi Province, Sumatra, Indonesia and was conducted within the framework of the interdisciplinary project “Ecological and socioeconomic functions of tropical lowland rainforest transformation systems (Sumatra, Indonesia)” - EFForTS. For more details on the study region and the experimental design of the project see Drescher et al. (2016).

Mites were extracted from soil of oil palm plantations in the vicinity of Bukit Duabelas National Park, Jambi Province, Sumatra, Indonesia. Soil samples (1–3 cm depth) were taken using a spade and each consisted of a core of 16 × 16 cm area. Mites were extracted from samples using the high gradient canister method described in Kempson et al. (1963). All specimens were collected in November 2013 by B. Klarner. Mites were stored in 70% ethanol until slide-mounting in Hoyer’s medium. For each mite, the gnathosoma was separated from the idiosoma and mounted next to it on the same slide.

Photographs and measurements were made using an Axiolab 5 phase contrast Zeiss microscope with an Axiocam 105 HD digital camera and Nikon Eclipse Ci connected to a computer-controlled digital camera Sight Ds-L3. Stacks of images were taken for each mite, using manual control of the focal plane. Selected images were combined using Zerene Stacker, version 1.04 (Zerene Systems, LLC 2009-2014). In some cases, images captured from different regions of the body were combined using the ‘photomerge’ function in Adobe Photoshop, version 2015 (16.0 or 20150529.r.88; Adobe Systems Inc., San Jose, USA). Digital drawings were prepared with Adobe Illustrator, version CC 2015 (19.0.0), based on (combined) photographs.

All measurements are given in micrometres (μm) and include the range (minimum–maximum). Lengths of shields were measured along their midlines and widths at their widest point, except for the sternometasternal shield which was measured at the level of insertion of setae *st2* and genitiventrianal shield between bases of *JV1*–*2*. Leg measurements were taken from the proximal margin of the coxa, along the midline of each segment, to the tip of the claw. Notations of body structures and idiosomal chaetotaxy follow Lindquist and Evans (1965) as adapted by Moraza and Peña (2006) and Marchenko (2016). Leg chaetotaxy follows Evans (1963) and Evans and Till (1965) and palps Evans and Till (1963). Idiosomal and peritrematal shield notations for pore-like structures (gland pores and poroids/lyrifissures) follow the system of Athias-Henriot (1971) for the ventral idiosoma and Athias-Henriot (1975) for the dorsal idiosoma.

Specimens of examined *K. angustatus* comb. nov. are deposited at NSMT (National Science Museum Natural History), Tokyo, Japan, the Holotype and voucher specimens at ESALQ (Escola Superior de Agricultura Luiz de Queiroz - Universidade de São Paulo), São Paulo, Brazil. Other collected materials are deposited at LIPI (Indonesian Institute of Science), Cibinong, Indonesia; SMNG (Senckenberg Museum), Görlitz, Germany; OSAL (Ohio State Acarology Collection), Columbus, USA and in ANIC (Australian National Insect Collection) Canberra, Australia. Additional photos of the species are digitally deposited in the online database Ecotaxonomy, accessible at http://www.ecotaxonomy.org.

The updated diagnosis and description of the genus were prepared after consulting the original description of the genus *Krantzolaspina* (Datta and Bhattacharjee 1989), as well as species descriptions (Metwali 1983, Ishikawa 1987a, Datta and Bhattacharjee 1989, Hajizadeh et al. 2017).

## Taxonomic accounts

### Parholaspididae

#### Genus Krantzolaspina

Taxon classificationAnimaliaMesostigmataParholaspididae

Datta & Bhattacharjee

07C1C085-A989-52BD-BF3E-1E85096A10EA

Krantzolaspina Datta & Bhattacharjee, 1989: 411.

##### Type species.

*Krantzolaspina rebatii* Datta & Bhattacharjee, 1989.

##### Diagnosis.

***Female*.** Dorsal shield entire, usually bearing 32–36 pairs of setae. Presternal area with two pairs of free presternal platelets. Sternometasternal shield well-defined, bearing four pairs of setae. Genitiventrianal shield, bearing four pairs of setae: one pair of genital setae *st5* and three pairs of preanal setae (*Zv1* and *Jv1–2*) in addition to circumanal setae. Podal-peritrematal shield free from genitiventrianal shield. Epistome with long median projection and with lateral margins serrate. Cheliceral movable digit with a pair of unequal plumose arthrodial brushes and no arthrodial corona or with a single plumose arthrodial brush and a fringed arthrodial corona. Palp trochanter with a small spur-like process near its ventral base (not palpfemur as in the original description); palptarsal claw three-tined. Pretarsus I reduced or absent, pretarsi II–IV well-developed. Chaetotaxy of femur-genu-tibia of legs I: 13-12-12; II: 11-11-10; III: 6-8-8; IV: 6-8-8.

***Male*.** Unknown.

##### Description.

***Female*.***Dorsal idiosoma*. Dorsal shield 340–561 μm long, broad anteriorly, gradually tapering posteriorly, anterior margin almost straight/truncate, partially covering idiosoma, leaving with the lateral and posterior margins of the soft integument broadly or narrowly exposed; shield mostly reticulate. Dorsal shield hypotrichous, bearing 32 (*J5* and *S5* slightly pilose in *K. angustatus* comb. nov.) or 36 pairs of smooth setae, most setae long and of similar length. Unsclerotised cuticle with 10–15 pairs of *r*, *R* and/or *UR* setae combined, smooth and moderately long (*UR* setae slightly pilose in *K. angustatus*).

*Ventral idiosoma*. All setae aciculate, smooth and relatively long (except *Jv4*–*5*, *Zv4*–*5* pilose in *K. angustatus*). Tritosternum with a pair of free pilose laciniae. Presternal area with two pairs of free, well-sclerotised presternal platelets. Sternometasternal shield well defined, longer than wide. Shield with posterior and anterior margin concave, partially smooth, reticulate or punctate (anterior and lateral margins ornamented in *K. angustatus*), bearing four pairs of setae *st1*–*4* and three pairs of poroids *iv1*–*3*. Peritrematal shield well-developed, broad, anteriorly free and posteriorly fused with developed podal shield; two pairs of poroids (*id7*, *ip*) and one pair of gland pores (*gdp*) posterior to stigma; Genitiventrianal shield longer than wide, reticulate, flask-shaped; cribrum developed. Soft opisthogastric cuticle with 6–7 pairs of setae; never hypertrichous. Metapodal platelets present or absent.

*Gnathosoma*. Subcapitulum with the corniculi well sclerotised, elongated and horn-like. Internal malae bifurcate, well separated from each other, densely fimbriated on outer margin and with apices slightly shorter than corniculi. Deutosternal groove with multidentate transverse rows, subcapitular setae smooth and aciculate, *h1*–*h3* often longer than *h2* and *pc.* Surface posterolaterad to seta *pc* with a pointed spine-like process or absent (simply flat). Epistome with a median projection and lateral margins irregularly serrate or may be smooth. Chelicera chelate-dentate; movable digit often bearing two teeth. A setiform pilus dentilus; smooth dorsal cheliceral seta, dorsal lyrifissure, a pair of unequal plumose arthrodial brushes or one plumose arthrodial brush and a narrow fringed arthrodial corona at base of the movable digit are present. Palp trochanter with a small pointed spine-like process in the ventral surface and with setae *v1* and *v2* slightly thickened and long, in contrast with setae of the other palp segments; palp tarsal claw three-tined.

*Legs* chaetotaxy as in diagnosis (see above).

***Male.*** Unknown.

### Re-description

#### Krantzolaspina angustatus

Taxon classificationAnimaliaMesostigmataParholaspididae

(Ishikawa, 1987) comb. nov.

BDCE6B4D-4C1A-5EED-95E1-5FEC2B8DB383

Proparholaspulus angustatus – Ishikawa, 1987a: 82; Kadkhodae et al., 2013: 131.
Indutolaelaps jiroftensis Hajizadeh et al., 2017**syn. nov.**

##### Diagnosis

**. *Female.*** Dorsal shield entire, mostly reticulate (except the anteromedial region which is smooth), partially covering the idiosoma, broad anteriorly and posteriorly narrowing; shield bearing 32 pairs of setae, most setae moderately long and smooth, except *j1*–*2* and *z1* which are slightly shorter and setae *S5* and *Z5* slightly pilose. Unsclerotised lateral cuticle of the idiosoma with a total of 15 pairs of smooth setae of similar length, except five pairs of *UR* setae slightly pilose, the pairs of setae *r6* and *R1* slightly shorter than *r5*–*7* plus seven pairs of *UR* setae. In the idiosoma ventre, all setae aciculate and smooth, except *JV4*–*5* and *ZV4*–*5* which are pilose. Presternal area with two pairs of free and presternal platelets. Sternometasternal shield mostly reticulate and covered by semi-rounded cells in the lateral margins, bearing four pairs of setae. Peritrematal shield anteriorly free, fused to the sternometasternal shield between coxae I–III, posteriorly fused with the parapodal shield and extended beyond posterior margin of the coxae IV, region of peritrematal + parapodal shield reticulate and covered by some semi-rounded cells; peritreme extending between coxae I–II at level of *st1*. Genitiventrianal shield longer than wide, reticulate, flask-shaped and bearing four pairs of setae *st5*, *JV1–3*, as well as three circumanal setae *pa* and *po.* Soft opisthogastric ventral cuticle with six pairs of setae *JV4*–*5*, *ZV2*–*5*. Metapodal platelets free, small and elliptical in shape. Deutosternal groove with six transverse rows, surface of the gnathosoma with pointed spine-like process similar to the ventral surface of the palp trochanter. Epistome with median projection bifurcate or trifurcate distally, lateral edges finely and irregularly serrate. Cheliceral digit movable and fixed with four and two teeth, respectively, base of movable digit with a plumose arthrodial brush and a narrow fringed arthrodial corona. Palp tarsal claw three-tined. Pretarsus I reduced or absent and pretarsi II–IV with pretarsi well-developed.

***Male*.** Unknown.

**Figure 1.**
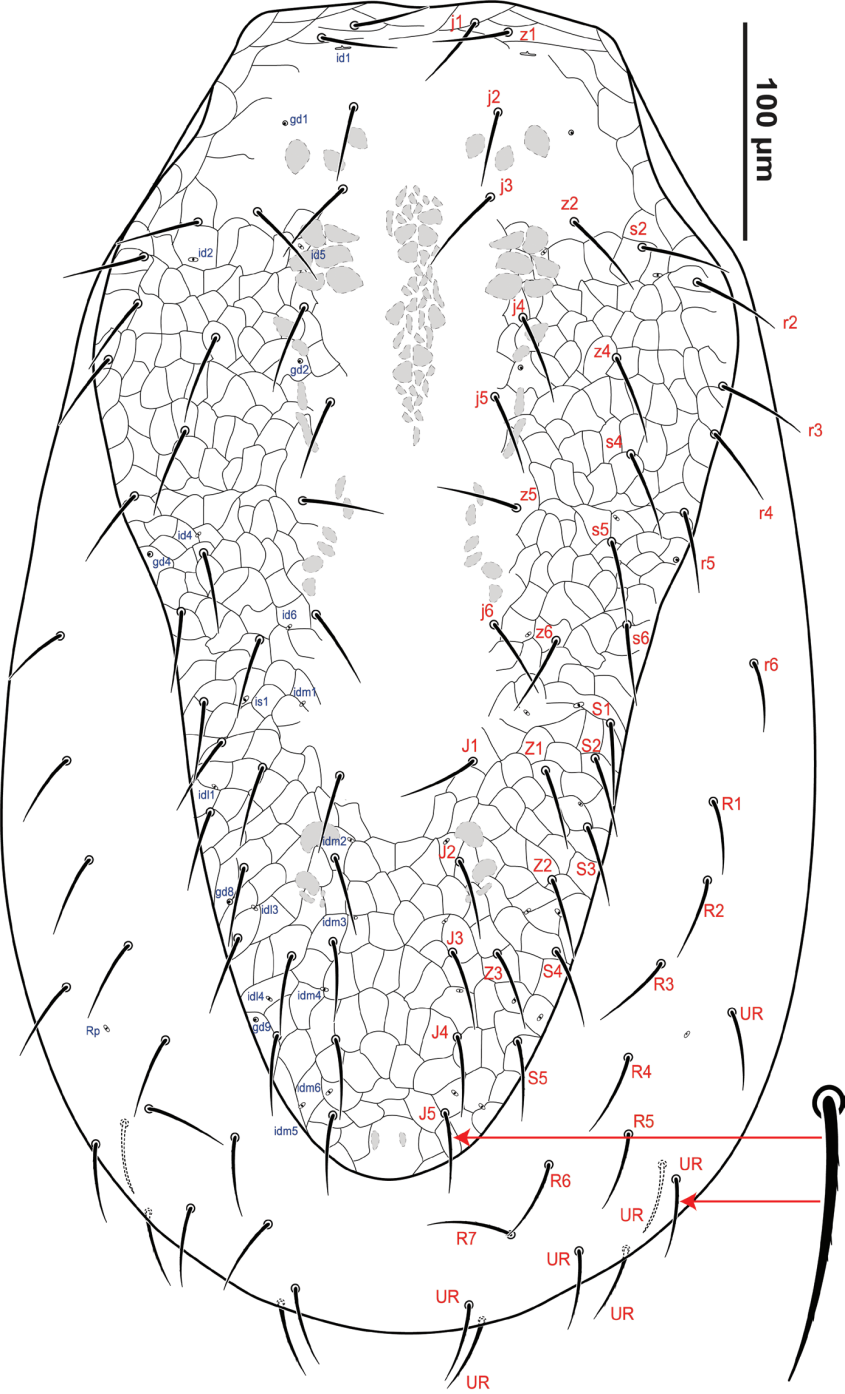
*Krantzolaspina angustatus* comb. nov., adult female. Dorsal idiosoma.

**Figure 2.**
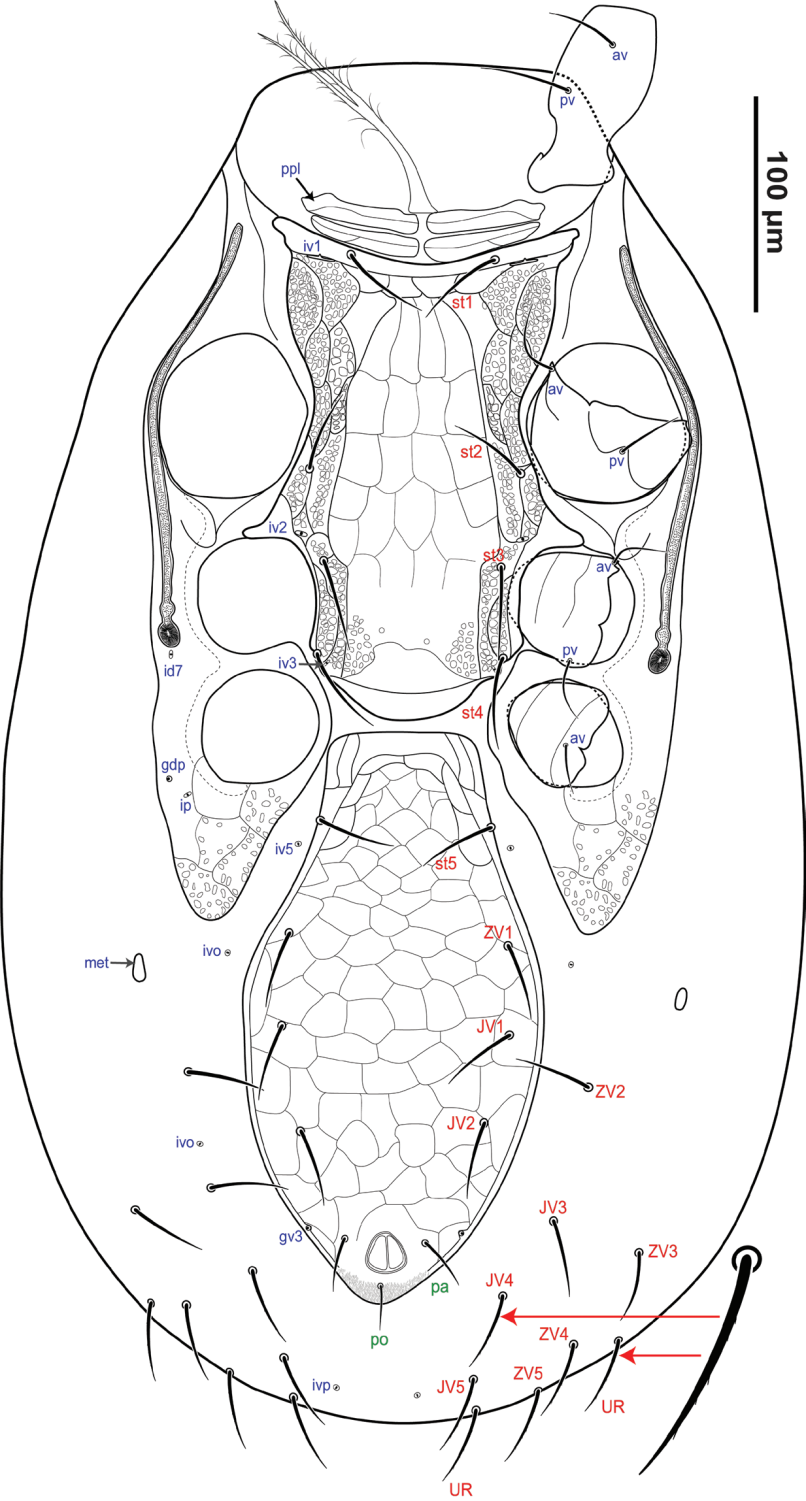
*Krantzolaspina angustatus* comb. nov., adult female. Ventral idiosoma.

**Figure 3.**
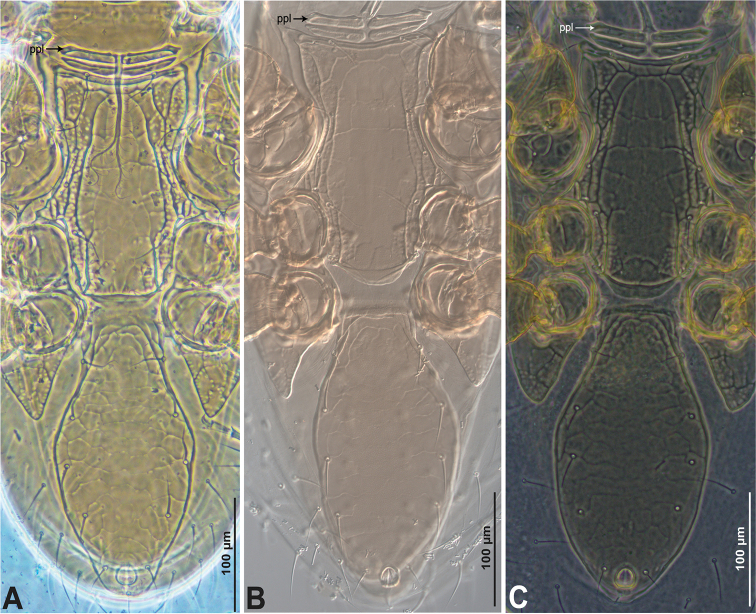
*Krantzolaspina angustatus* comb. nov., adult female. Ventral idiosoma showing the sternometasternal, genitiventrianal and parapodal shield. **A** Holotype of *Krantzolaspina angustatus***B** Paratype of *Indutolaelaps jiroftensis* syn. nov., photos by Raphael Castilho **C** New material from Indonesia.

**Figure 4.**
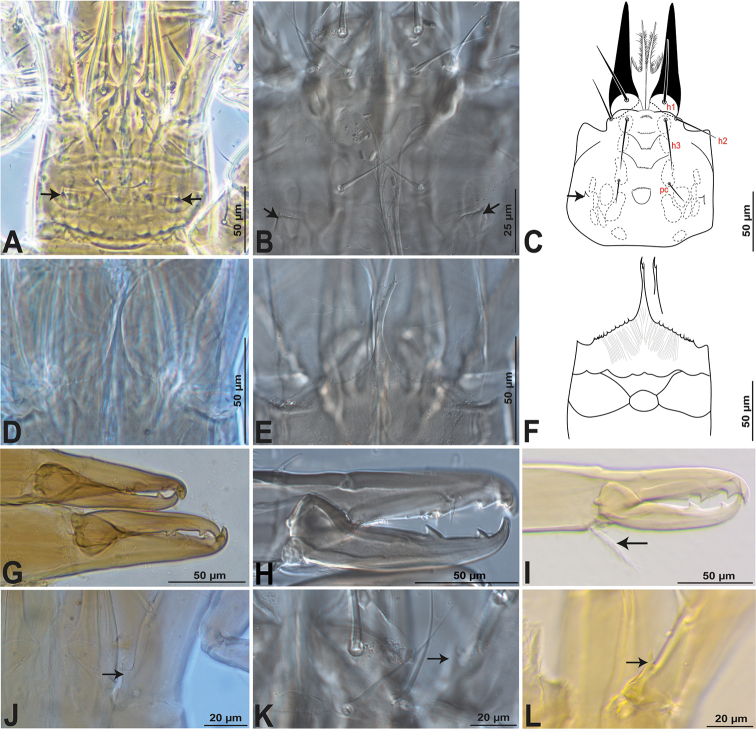
*Krantzolaspina angustatus* comb. nov., adult female, Gnathosomal structures. Holotype (Left); *Indutolaelaps jiroftensis* syn. nov. (Middle), photos by Raphael Castilho; New material from Indonesia (Right). **A–C** Subcapitulum **D–F** Epistome **G–I** Chelicera **J–L** Palp trochanter with a ventral spine-like process.

**Figure 5.**
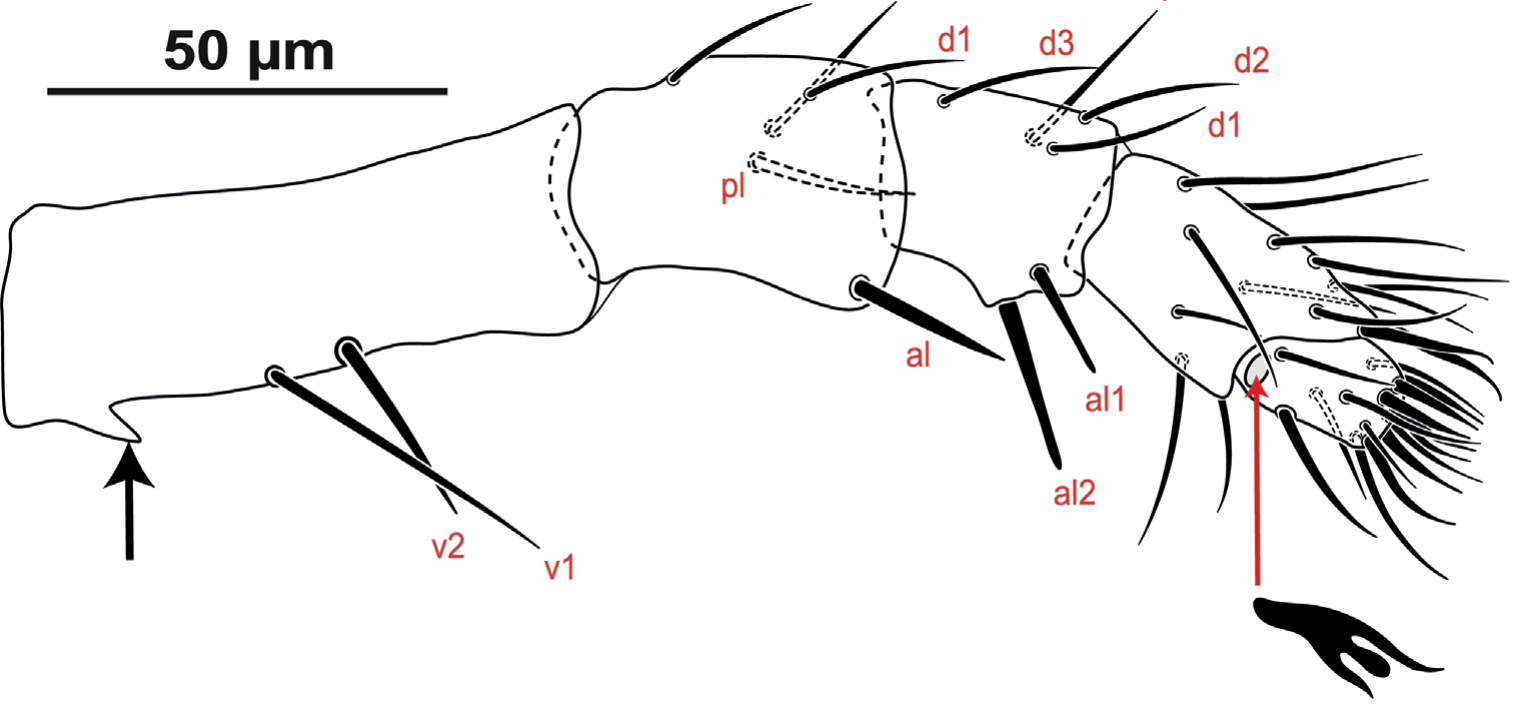
*Krantzolaspina angustatus* comb. nov., adult female. Palp, note the ventral spine-like process in palptrochanter.

**Figure 6.**
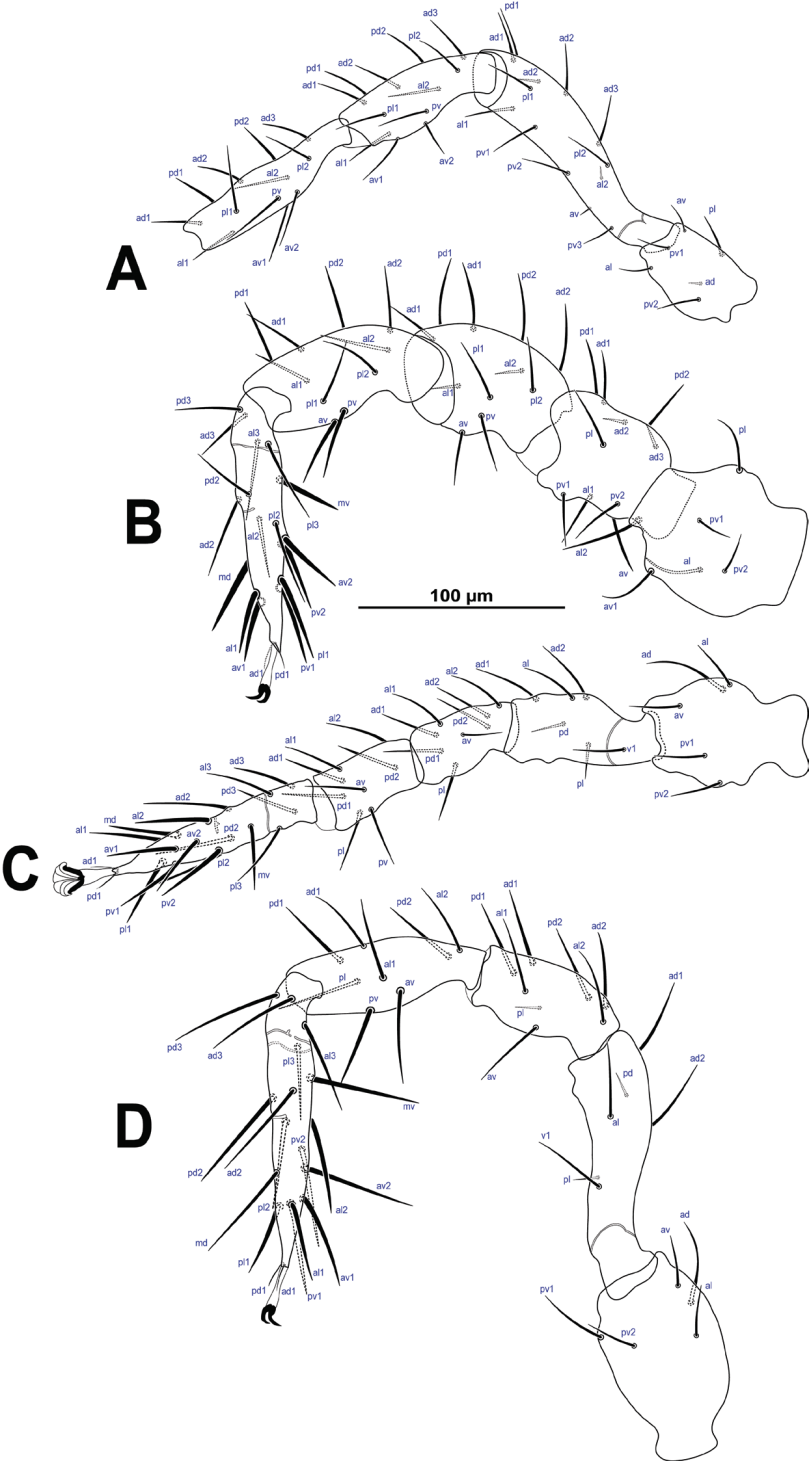
*Krantzolaspina angustatus* comb. nov., adult female. **A–D** Legs I–IV, respectively. Coxae I–IV in the Fig. 2.

***Female*** (Figs 1–6) (*n = 6*). *Idiosomal dorsum* (Fig. 1). 541–611 μm long, 287–372 μm wide. **Dorsal shield** 514–536 μm long, 219–239 μm wide (at level between *r2*–3), entire and broad anteriorly with the anterior margin slightly straight and posteriorly narrowing, covering partially the idiosoma with the lateral and posterior margins of soft integument broadly exposed, most surface of the shield reticulate, but smooth on the dorsocentral region between the bases of *z1* and *J2*. Dorsal shield bearing 32 pairs of setae of similar length and shape, most setae relatively long (36–46 μm) and smooth, except *j1*–*2* and *z1* slightly shorter (31–35 μm) and the setae *S5* and *Z5* slightly pilose (Fig. 1). **Podonotal region** with 17 pairs of setae *j1*–*6*, *z1*–*2*, *z4*–*6*, *s2*, *s6*, *r2*–*5* and 10 pairs of poroids, including three pairs of glands *gd1*–*2* and *gd4*. **Opisthonotal region** with 15 pairs of setae *J1*–*5*, *Z1*–*5*, *S1–5* and 10 pairs of poroids, including two glands *gd8* and *gd9*. **Unsclerotised lateral cuticle** bearing a total of 15 pairs of smooth setae of similar length (38–44 μm) except five pairs of *UR* setae slightly pilose (Fig. 1); the pairs of setae *r6* and *R1* slightly shorter than *r5*–*7* and the six pairs of *UR* setae (two of them on the ventral side); a pair of lyrifissures (*Rp*) present between *R3* and *R4.*

*Idiosomal venter* (Figs 2, 3A–C). All setae aciculate and smooth, except *Jv4*–*5*, *Zv4*–*5* which are pilose (Fig. 2). **Tritosternum** (Fig. 2) with columnar base and pair of free pilose laciniae. **Presternal area** bearing two pairs of free, well-sclerotised, transversely aligned and presternal platelets *ppl* (Figs 2, 3A–C), with transversal lineae over surface. **Sternometasternal shield** (Figs 2, 3A–C) 202–209 μm long, 104–110 μm wide (at level of *st2*) well-defined, reticulate and covered by semi-rounded cells in the lateral margins, posteriomedial area smooth beyond to the setae *st3*; anterior and posterior shield margin concave, bearing four pairs of setae *st1*–*st4* (36–41) and three pairs *iv1*–*3* of slit-like poroids; *iv1* larger than *iv2*–*3*, the latter rounded. **Peritrematal shield** (Fig. 2) broad, anteriorly free, fused to the exopodal shield (distinctly more sclerotised), to sternometasternal shield between the coxae I–III and posteriorly with the well-developed parapodal shield; shield extended beyond the posterior margin of the coxa IV, this area reticulate and covered by some semi-rounded cells; two pairs of poroids (*id7*, *ip*) and one pair of gland pores (*gdp*) discernible. Peritreme extending anteriorly between coxae I–II, at level of seta *st1*. **Genitiventrianal shield** (Figs 2, 3A–C) 246–264 μm long × 127–135 μm wide, reticulate and flask-shaped; shield with four pairs of setae *st5* 35–37 (slit-like poroids *iv5* on unsclerotised cuticle and posterolaterad of *st5*) *ZV1*, *JV1*–*2* 39–42 additionally to the circumanal setae *pa* and *po*, paranal setae *po* (27–29) aligned with anterior margin of anal opening, postanal seta shorter *po* (15–17); gland opening *gv3* on posterolaterad shield margins, at level slightly anterior to paranal setae, cribrum well-developed. Soft opisthogastric cuticle with seven pairs of setae *JV3*–*5*, *ZV2*–*5* (39–40) and three poroids, including one (*ivp*). **Metapodal** (Fig. 2; *met*) platelets free, small and suboval.

*Gnathosoma* (Fig. 4A–L). **Subcapitulum** (Fig. 4A–C): corniculi well sclerotised, elongated and horn-like slightly shorter than cheliceral movable digit. Internal malae bifurcate, totally separated from each other, densely fimbriated on outer margin and with apices slightly shorter than corniculi. Deutosternal groove with six transverse rows denticles, each bearing 8–21 denticles and distal row smooth, with lateral ridges each side of the 2^nd^ and 3^rd^ row; subcapitular setae smooth and aciculate, *h1* 51–54 and *h3* 40–43 longer than *h2* 34–37 and *pc* 17–20. Surface posterolaterad to seta *pc* with a minute or a small spine-like process (Fig. 4A–C). Epistome (Fig. 4D–F) with a median projection bifurcate or trifurcate distally, lateral edges finely and irregularly serrate. **Cheliceral** (Fig. 4G–I) fixed digit (106–111) with four teeth (most proximal small and most distal inserted subapically) plus one distal hook-like tooth and a setiform *pilus dentilus*; dorsal and anti-axial lyrifissures present as well as dorsal setae smooth; movable digit (97–103) with two teeth directed backwards and one distal hook-like tooth, base of the movable digit with a plumose arthrodial brush (44–48) (Fig. 4I, see arrow) much shorter than movable digit and a narrow fringed arthrodial corona which is only discernible ventrally. **Palp** (Fig. 5) with normal chaetotaxy for Parholaspididae, with 2-5-6-14-15 setae on trochanter-femur-genu-tibia-tarsus; palptrochanter almost twice longer than palpfemur, with a small pointed spine-like process on the ventral surface (Figs 4J–L, 5; see arrow) and setae *v1*–2 thickened and long, in contrast to the setae of the other palp segments; palpfemur *al* thickened, palpgenu with setae *al1*–2 thickened and spatulate distally. Palp tarsal claw three-tined, medial tine conspicuous distally spatulate.

*Legs* (Fig. 6A–D) lengths (in μm): I (Fig. 6A): 573–625, II (Fig. 6B) 461–495, III (Fig. 6C) 396–421, IV (Fig. 6D) 550–575. The leg chaetotaxy/setation—Coxae: I 2 (0, 0/1, 0/1, 0); II: 2 (0, 0/1, 0/1, 0); III: 2 (0, 0/1, 0/1, 0); IV: 1 (0, 0/0, 0/1, 0); trochanters: I: 6 (1, 0/1,1/2, 1); II: 5 (1, 0/1, 0/2, 1); III: 5 (1, 1/1, 0/2, 0); IV: 5 (1, 1/1, 0/2, 0); femora: I: 13 (2, 3/1, 2/3, 2); II: 11 (2, 3/1, 2/2, 1); III: 6 (1, 2/1, 1/0, 1): IV: 6 (1, 2/1, 1/0, 1); genua: I: 12 (2, 3/2, 2/1, 2); II: 10(2, 2/1, 2/1, 2); III: 8 (2, 2/1, 2/1, 1); IV: 8 (2, 2/1, 2/0, 1); tibia: I: 12 (2, 3/2, 2/1, 2); II: 10 (2, 2/1, 2/1, 2); III: 8 (2, 1/1, 2/1, 1); IV: 8 (2, 1/1, 2/1, 1); tarsi I: not counted, II: 18; III: 18; IV: 18. Pretarsus I reduced or absent and pretarsi II–IV well-developed, including a pair of claws and a pulvillus. Legs with all setae aciculate and smooth, most setae are relatively long, except leg III which has comparatively shorter setae, tarsi I–IV which have longest and thicker setae than leg segments especially tarsus IV and a set of very short setae as follows: *ad* in trochanter I, *av* and *ad_2_* and *al_2_* in femur I, *ad_2_*–*_3_* in femur II, *al_1_*–*_2_* in genu II, *pl* and *pd* in femur IV and *pl* in genu IV.

***Male.*** Unknown.

##### Material examined and depository.

• 1♀ Holotype at NSMT (NSMT-Ac 9805) collected in Philippines, Palawan Islands, Olanguan Valley, between Puerto Princesa and Roxas, on litter of tropical forest. •16♀ vouchers at ESALQ collected in Iran, Kerman Province on soil and litter at the base of *Medicago sativa* (Fabaceae) and *Citrus sinensis* (Rutaceae). New material from Indonesia, Sumatra, Jambi Province, Bukit Duabelas region, upper soil layer (0–3 cm) of oil palm plantation, research site BO2b, 2°04'32.0"S, 102°47'30.7"E, 83.74 m a.s.l. 6♀ on slides as follows: • 1♀ deposited at LIPI (OSAL 00124840); • 1♀ deposited at SMNG (OSAL 00124839 SMNG 2020/62099). • 2♀ deposited at OSAL (OSAL 00124841, 00124842). • 2♀ deposited at ANIC (OSAL 00124843, 00124844). 10♀ in alcohol, as follows: • 2♀ deposited at LIPI, • 3♀ deposited at SMNG, • 2♀ deposited at OSAL and • 3♀ deposited at ANIC. All specimens were collected in November 2013 by B. Klarner. Additional photos of the species are deposited in Ecotaxonomy database at www.ecotaxonomy.org (ECOTAX_ID: 434549).

##### Differential diagnosis.

*K. angustatus* comb. nov. significantly differs from *K. rebatii* and *K. solimani* comb. nov. in the following combination of characters: *K. angustatus* has 32 pairs of smooth dorsal setae (except *S5* and *Z5* slightly pilose), while *K. rebatii* and *K. solimani* have 36 pairs of smooth setae, respectively; *K. angustatus* has 15 pairs of setae *r*-*R* and *UR*-series in the unsclerotised lateral cuticle, whereas *K. solimani* and *K. rebatii* have 13 and 10, respectively; in *K. angustatus* and *K. solimani*, the peritreme is extended anteriorly between coxae I–II at the level of *st1*, while in *K. rebatii*, it is extended beyond coxa I; in *K. angustatus*, a spine-like process is developed in the subcapitulum, while this is absent in *K. rebatii* and *K. solimani*. Further, the arthrodial process in *K. rebatii* has a pair of long unequal arthrodial brushes, whereas the arthrodial process in *K. solimani* and *K. angustatus* has a relatively long arthrodial brush and a narrow fringed arthrodial corona. Additionally, in *K. angustatus*, small metapodal platelets elliptical in shape are present, while in *K. solimani* and *K. rebatii*, they are absent. Lastly, Table 1 provides uncertain/unclear or unknown characters of these species.

**Table 1.** Characteristics of the females of *Krantzolaspina angustatus* comb. nov., and some ambiguous or unknown data of *K. rebatii* and *K. solimani* comb. nov.

**Characters**	***K. angustatus* (Ishikawa, 1987) comb. nov.**	*K. rebatii Datta & Bhattacharjee, 1989	*K. solimani (Metwali, 1983) comb. nov.
Dorsal shield ornamentation	mostly reticulate (smooth on the dorso-central region around bases of *z1* and *J2*)	completely smooth?	with tetra- and pentagonal reticulation (except on dorsocentral region of setae *j–J*, faintly ornamented)
Sternometasternal / ventrianal shield ornamentation	completely reticulate and with the lateral margins covered by some semi-rounded cells	mostly smooth but with lateral margins pointed?/ anterior margin pointed, elsewhere smooth?	mostly with tetra and pentagonal reticulation
Parapodal-peritremal shield ornamentation	slightly reticulate + semi-rounded cells posterior to the coxa IV	completely smooth?	completely smooth?
**^1^**Setae of opisthogastric cuticle (*JV*–*ZV*)	Seven pairs (*JV3*–5, *ZV2*–*5*); *JV4*–*5* and *ZV4*–*5* pilose	Six pairs (*JV4*–*5*, *ZV2*–*5*)?	Six pairs (*JV4*–5, *ZV2*–*5*)
Deutosternum: no. rows	Nine	three?	?
Leg chetotaxy			?
(Coxa – Tibia)			
I	2, 6, 13, 12, 12, not counted	?, ?, 10, 11, 11, ?	
II	2, 5, 11, 10, 10, 18	?, 4, 10, 9, ?, ?	
III	2, 5, 6, 8, 8, 18	?, 3, 5, 8, 7, ?	
IV	1, 5, 6, 8, 8, 18	?, ?, 4, 6, 7, ?	

? indicates unknown or uncertain/unclear data. **^1^**All setae are smooth and moderately long, except when mentioned otherwise. * holotype not found, presumed lost; characters presented are based on the original description.

##### Remarks.

*Krantzolaspina* is a well-defined genus by the unique combination of characters stated above (see diagnosis of the genus). However, a number of characters are also present in other dermanyssine families, for example, well-developed arthrodial brush(es) is/are also present in macrochelid mites, a sternometasternal shield bearing *st1*–*st4* is shared with species of Pachylaelapidae (Mašán and Halliday 2014) and Ologamasidae (Castilho et al. 2016) and the fusion of genital + ventral + anal shields forming a genitiventrianal shield is shared with two genera of Laelapidae (*Ololaelaps* and *Oloopticus*) (Beaulieu et al. 2019).

Although the genitiventrianal shield is a diagnostic character for *Krantzolaspina*, it is not an exclusive character as it also occurs in other genera of Parholaspididae, such as *Holaspulus*, some species of *Holaspina* and *Proparholaspulus*, as well as in other families, such as Leptolaelapidae genus *Indutolaelaps* Karg, 1997. In addition, this feature occurs in some species of Laelapidae (see above), but in those species, the genitiventrianal shield is hyper-developed posteriorly and occupies most of the opisthogaster and is often named the hologastric shield (Beaulieu et al. 2019).

One particular feature of *Krantzolaspina* is the pointed spur-like process present on the palp trochanter and this character is important for recognising the genus. However, a similar process is present in species (and all post-embryonic stages) of the monotypic family Megalolaelapidae (*Megalolaelaps*), in which the palp trochanter typically bears a large anteroventral horn-like projection. Unfortunately, the function of these processes of the palp trochanter in *Krantzolaspina* and *Megalolaelaps* is unknown (Mašán and Halliday 2014, Cómbita-Heredia et al. 2018). It should be noted that members of Megalolaelapidae are more similar morphologically to Pachylaelapidae and to Macrochelidae of the genus *Neopodocinum* Oudemans (Macrochelidae) than to Parholaspididae (Cómbita-Heredia et al. 2018).

### Distribution of *Krantzolaspina* spp.

*Krantzolaspina* species have been collected from soils, mainly disturbed soil and in countries of Southern Asia. However, its distribution is isolated since they are present in Iran, Philippines and recently recorded in Indonesia (see details of locality type and other records, Table 1). A similar pattern of isolated distribution is present in other species of the parholaspidid family (e.g. *Holaspina alstoni* and *Parholaspis kewensis*, *P. meridionalis*) which were collected in botanical gardens from England where these records were based on accidental introduction (Ishikawa 1980c, Latifi et al. 2006) and potentially facilitated by human activity (Latifi et al. 2006). Hypothetically, we believe that these records of *Krantzolaspina* are based on introduced specimens through the trading of vegetal material including soil where they inhabit.

### *Notes on Indutolaelaps jiroftensis*Hajizadeh et al., 2017 syn. nov. of *K. angustatus* (Ishikawa, 1987) comb. nov.

This species was described in the genus *Indutolaelaps* Karg, 1997 (Leptolaelapidae), based on a genitiventrianal shield, an epistome with an anteriomedial extension, wider at the base and acute distally and a palp tarsal claw three-tined (Hajizadeh et al. 2017). The misplacement of *I. jiroftensis* in the genus *Indutolaelaps* may suggest some genus-level similarity of *Indutolaelaps* and *Krantzolaspina*. These two genera indeed share several conspicuous characters such as similar shape of the genitiventrianal shield, presence of two pairs of presternal platelets, sternometasternal shield bearing *st1*–*4*, parapodal shield well-developed and fused with peritrematal shield, epistome with a narrow median projection and lateral margins serrate and 3-tined palptarsal claw. However, both genera which belong to different families can be distinguished by the combination of characters given in Table 2, such as arthrodial process, number of presternal platelets and preanal and dorsal setae.

**Table 2.** Morphological characteristics of females in the genera *Krantzolaspina* and *Indutolaelaps*.

Morphological characters	*Krantzolaspina Datta & Bhattacharjee, 1989 (Parholaspididae)	**Indutolaelaps Karg, 1997 (Leptolaelapidae)
Arthrodial process	with two arthrodial brush or one arthrodial brush and one narrow fringed arthrodial corona	one fringed arthrodial corona
Presternal platelelets	two pairs	one pair
Number of preanal setae in the genitiventrianal shield	three pairs	five pairs
Number of dorsal setae	32–34 pairs	50–55 pairs
Gnathotectum	with a median projection slightly bifurcate, trifurcate or serrate distally and with lateral margins serrate	with a median projection, distally and lateral margins smooth
Deutosternal rows	six, nine	five
Cheliceral dentition	FD usually with four teeth (rarely three) and MD bidentate	FD with three teeth and MD bidentate
Pretarsi	pretarsus I reduced or absent, pretarsi II–IV well developed	Pretarsus I reduced, pretarsi II–IV well developed

The present differential characters are listed in order of importance. **^1^**All setae are smooth and moderately long, except when mentioned otherwise. * based on the present review; ** based on the original description of Karg (1997). FD – fixed digit; MD – mobile digit.

Further, the synonymy of *I. jiroftensis* is supported in that it has 32 pairs of dorsal setae, two pairs of presternal platelets, sternometasternal and genitiventrianal shield with four pairs, as well a distinct ornamentation pattern (Hajizadeh et al. 2017 pp 670–671), which are diagnostic characters of the genus *Krantzolaspina* and specifically of *K. angustatus*. Additionally, the characters present in their drawings (figs 3, 5–7) and our photos (Figs 3B, 4B, E, H, K) of *I. jiroftensis* (Hajizadeh et al., 2017) match the characters from the holotype of *K. angustatus* (see Figs 3A, 4A, D, G, J).

### Notes on *Krantzolaspina rebatii* Datta & Bhattacharjee, 1989

*Krantzolaspina rebatii* Datta & Bhattacharjee, 1989: 411

The holotype of *K. rebatii* was deposited according to Datta and Bhattacharjee (1989) in “Collection of Animal Ecology Laboratory, Department of Zoology, Gauhati University, Guwahati, India”; however, we were unable to locate this type specimen despite significant efforts. A careful study of type material will be essential to identify the diagnostic traits of that species.

Focusing on the original description, we like to mention some discrepancies and/or mistakes that we found between the text and illustrations as follows:

(1) *Dorsal shield*: “36 pairs of setae” is indicated in the text (Datta and Bhattacharjee 1989:411); however, their illustration (fig. 1b) shows only 34 apparent pairs of setae, although five represented only by sockets (presumably because the setae had fallen off). In addition, it is unclear whether the dorsal shield is smooth or not, as any type of ornamentation seems to be excluded from their original drawings and text.

(2) *Venter*: The ornamentation seems to be excluded from their original text, but it looks mostly smooth in fig. 1a of Datta and Bhattacharjee (1989) as well the lateral region punctate of the sternometasternal shield and the margin anterior of the genitiventrianal shield.

(3) *Legs*: Datta and Bhattacharjee (1989) provided an illustration of the legs (Fig. 1i–h), but without accompanying text in the description. The illustrations indicate a reduced number of setae compared with the leg chaetotaxy herein described for *K. angustatus* (Table 1). We presume that some setae were overlooked and not drawn by Datta and Bhattacharjee (1989).

### Notes on *K. solimani* (Metwali, 1983) comb. nov.

*Neoparholaspulus solimani* Metwali, 1983: 459.

Metwali (1983) placed this species in the genus *Neoparholaspulus*, based on some characters that are typical for the genus, such as a genitiventrianal shield and one pair of presternal platelets, as well as one pair of metasternal shields free. In addition, Metwali’s description of the species includes “metasternal plate well developed and free”. However, the description also states that the sternal shield has four pairs of setae and the illustration shows that the metasternal plates are fused to the sternal shield. We have provisionally placed this species in *Krantzolaspina*, based on the presence of 32 pairs of setae in the dorsal shield, two pairs of presternal platelets and the assumption that fig. 2 of Metwali (1983) is inaccurate and that the metasternal plates are indeed fused to the sternal shield. Unfortunately, as the type specimens are lost, this interpretation cannot be confirmed (Reham Abo-Shnaf, personal communication).

### *Krantzolaspina* sp.?

Nawar and El-Sherif (1995: 273) re-described the female of a species that they identified as *Holaspina solimani* and described the male for the first time. Hussein et al. (2002: 1117) reared this species in the laboratory and studied its biology and behaviour. However, the illustrations and description in Nawar and El-Sherif (1995) differ in the grade of fusion of the metasternal plate, as well as the number of setae in the sternal shield from those in Metwali (1983) and we provisionally assume that these two specimens are two different species. Unfortunately, the specimens examined by Nawar and El-Sherif (1995) and Hussein et al. (2002) are lost and this interpretation cannot be confirmed (Reham Abo-Shnaf, personal communication).

In conclusion, the genus *Krantzolaspina* currently includes three valid species *Krantzolaspina angustatus* (Ishikawa, 1987) comb. nov., *K. rebatii* Datta & Bhattacharjee, 1989 and *K. solimani* (Metwali, 1983) comb. nov. Lastly, despite the valuable work undertaken by Krantz (1960), Petrova (1967, 1970, 1977), Ishikawa (1980a, 1980b, 1987b), Datta and Bhattacharjee (1989, 1991), Lee and Lee (2000) and the more recent works by Bhattacharyya and Kheto (2016) for *Proparholaspulus* and by Marchenko (2016) for *Neparholaspis*, it is clear that a revision of the family Parholaspididae is needed to know the current status.

## Supplementary Material

XML Treatment for Krantzolaspina

XML Treatment for Krantzolaspina angustatus
